# Public health in the genomic era: will Public Health Genomics contribute to major changes in the prevention of common diseases?

**DOI:** 10.1186/0778-7367-69-8

**Published:** 2011-12-05

**Authors:** Evy Cleeren, Johan Van der Heyden, Angela Brand, Herman Van Oyen

**Affiliations:** 1 Operational Direction Public Health and Surveillance, Scientific Institute of Public Health, Brussels, Belgium; 2 Institute for Public Health Genomics (IPHG), Cluster of Genetics and Cell Biology, Faculty of Health, Medicine & Life Sciences, Maastricht University, Maastricht, Netherlands

**Keywords:** Epidemiology, Genomics, Epigenomics, Prevention, Public Health, Public Health Genomics, Translational Research, Policymaking, Personalised Healthcare

## Abstract

The completion of the Human Genome Project triggered a whole new field of genomic research which is likely to lead to new opportunities for the promotion of population health. As a result, the distinction between genetic and environmental diseases has faded. Presently, genomics and knowledge deriving from systems biology, epigenomics, integrative genomics or genome-environmental interactions give a better insight on the pathophysiology of common diseases. However, it is barely used in the prevention and management of diseases. Together with the boost in the amount of genetic association studies, this demands for appropriate public health actions. The field of Public Health Genomics analyses how genome-based knowledge and technologies can responsibly and effectively be integrated into health services and public policy for the benefit of population health. Environmental exposures interact with the genome to produce health information which may help explain inter-individual differences in health, or disease risk. However today, prospects for concrete applications remain distant. In addition, this information has not been translated into health practice yet. Therefore, evidence-based recommendations are few. The lack of population-based research hampers the evaluation of the impact of genomic applications. Public Health Genomics also evaluates the benefits and risks on a larger scale, including normative, legal, economic and social issues. These new developments are likely to affect all domains of public health and require rethinking the role of genomics in every condition of public health interest. This article aims at providing an introduction to the field of and the ideas behind Public Health Genomics.

## Introduction

Only 50 years after the discovery of the DNA structure by James Watson and Francis Crick, the scientific world completed the sequencing of the entire human genome, the Human Genome Project. As a consequence, the field of human genome research knew a rapid discovery phase with international initiatives like the "1000 Genomes project" or the "Personal Genome Project" and the development of the necessary technological tools [1]. Furthermore, there was a rise in the research field of the -omics: (epi)genomics, proteomics, interactomics, metabolomics, nutrinomics... These domains of systems biology gave rise to a better understanding of the human physiology and pathophysiology [2].

Although the terms 'genetics' and 'genomics' are sometimes used interchangeably, they have a different meaning. Genetics is the science of inheritance and focuses on Mendelian inherited traits where mostly a mutation in one gene causes the disease. Genomics is the study of a complex set of genes, their expression and how they interact with other genes and the environment to affect how a condition develops.

Classically, there is a distinction between environmental and genetic diseases. However, this point of view has changed. There is hardly any disease without a possible genetic contribution. The common diseases, or the complex diseases, are caused by an interplay of different genetic, lifestyle and environmental factors. In 2006 the Institute of Medicine published a report in which it pointed out a critical need for transdisciplinary research that would integrate genomics and the biological sciences with the behavioural and social sciences, thus joining 'nature' and 'nurture' [3].

Public health interventions have so far focussed on environmental-specific factors principally due to scientific and technological limits. The advances brought about by genomics are changing these perceptions. Exogenous influences will remain of vital importance for public health, but focussing merely on these influences may never equal the triumphs of the past [4]. To stay successful in promoting health and preventing disease, the effectiveness of public health interventions must be strengthened by incorporating all determinants of disease: the internal host-specific factors and their interactions with the environment [5].

In 2005, a multidisciplinary group of eighteen experts form Canada, France, Germany, the UK and the US reached a consensus about how to develop genomics optimally for the benefit of population health [6]. An agreed concept for the enterprise of translating genome-based science and technology into improvements in population health was developed and is extensively discussed elsewhere [6,7]. The experts defined Public Health Genomics (PHG) as "the responsible, effective integration of genome-based knowledge and technologies into health services and public policy for the benefit of population health". This article aims at giving an overview of the concepts of PHG, based on the elements of this definition. Thus, it will start to give an overview of the proposed steps to be taken to implement the results of basic science into public health benefits, followed by the role of epidemiology and the necessary epidemiological tools. Then it will continue by describing the use of genomics in current and future medical and public health practices and it concludes with an outline of the multidisciplinary character of PHG. The key issues of this article are highlighted in Table 1.

**Table 1:** Challenges in Public Health Genomics and what public health authorities can do about them?

*The ultimate goal of PHG* Once we understand both the genetic and environmental factors involved in the causation of a disease, and how they interact, it will be possible to devise effective preventive interventions targeted at strata of the population with specific genotypes	
*Challenges*	*Possible role of PH authorities*
Translational research	• Conducting systematic reviews/meta-analyses of reported genetic associations; • Development of evidence-based policy and practice guidelines; • Dissemination, implementation and diffusion of research; • Monitoring population health impact

Epidemiological tools	• Study how biobanks/disease registries can be extended with genomic information

Reconciling traditional public health concerns with effective health interventions tailored at the level of groups of individuals	• Serving as the honest broker for emerging genomic application in practice; • Implementing evidence-based genomic applications; • Preventing premature use, misuse and overuse of genomic applications; • Evaluating current public health interventions using genomic tools; • Elaborating appropriate methods to collect and monitor the genome-based research, identifying information gaps (population and individual level), formulating policy development; • Systematic evaluation in different populations and individuals; • Quantifiying the use of genetic tests and services, the quality of the tests used, and compare this with established standards and guidelines; • Considering to change the public health approach into looking at the individual first, then subpopulations, followed by the population as a whole

Ethical, legal and social issues	• Monitoring the implications of genome-based knowledge in a broad social context, thereby improving consumer protection; • Ensuring the confidentiality, privacy and autonomy of the population; • Establishing management pathways that describe the process of feedback of research information to people enrolled in biobanks/registries that uses genomic information; • Ensuring the proper education of first-line caregivers, physicians, researchers working with human genomics data and the general population, to ensure that valid information reaches the population; • Taking an active role in the transformation of current biobanks to genomic biobanks; • Participate actively in updating the informed consent process (proper counselling session, discussing the possible outcome for relatives, the possibility to withdraw from the study, international digital datasharing, feeding back research results...); • Encountering the possibilities of social inequalities in health, (possibly) introduced by genome-based knowledge; • Merging the competing interests of all different stakeholders into a collaborative framework that rewards both scientific innovations and appropriate clinical applications

## Translational research

The relation between a DNA sequence and a phenotype is far from simple. This means that the information about genes and DNA sequences must be translated into facts about genetic susceptibility to disease, the interaction between these susceptibilities and modifiable risk factors and the impact of this knowledge on population health. Khoury and co-workers presented a framework for the continuum of multidisciplinary translation research in genomic medicine [8]. The purpose is to move promising genomic applications to clinical and public health practice for population health benefit and consists of four phases: T1: gene discovery to candidate health application; T2: health application to evidence-based practice guidelines; T3: practice guidelines to health practice and T4: practice to population health impact [8].

T1 research starts with gene discovery. However, this discovery alone is not enough, it has to be evaluated how genomic discoveries can be used to develop promising health applications. These are used either in clinical evaluation or in selection of effective therapeutic options. An emerging example of T1 research is the construction of "genomic profiles", testing combinations of genetic variants to predict increased risk of common diseases and potentially guide interventions [9]. T1 research in genomics comprises both observational studies and clinical trials. Human genetic epidemiology plays a fundamental role in this kind of knowledge integration. The Human Genome Epidemiology Network (HuGENet) is a global collaboration of individuals and organisations to assess population health impact of human genomics variation and how it can be used to improve health and prevent disease in populations [10]. This means that, before clinical applications can be considered, all the accumulated knowledge about a set of gene variants must be carefully assessed, including prevalence in different populations, the strength of the association with different disease endpoints, and interactions between gene variants and social and environmental determinants of risk. This results in rigorous systematic reviews and meta-analyses of genetic associations, the HuGE reviews [11]. HuGENet further maintains a continuously updated knowledge base, called the HuGE Navigator which lists all the reported genetic associations with certain traits [12].

An important application of HuGE research is the evaluation of clinical validity of complex genetic tests which is embraced as T2 research [8]. Nowadays the development and evaluation of genomic applications for use in practice is an unregulated process. Translation of a genetic test from research into practice starts with the identification of the disorder to be tested for, the specific test to be used, and the clinical scenario in which the test will be used (e.g. for diagnostic versus predictive purposes and the population to be tested). The genetic test can then be evaluated in a four-step process represented by the acronym ACCE: analytic validity, clinical validity, clinical utility and ethical, legal and social implications [8]. Analytic validity is the ability to accurately and reliably measure the genotype of interest [13]. Genetic tests should have high sensitivity, specificity and predictive values which are measured by the clinical validity. For complex genotypes the clinical validity of genetic tests is not clear and is likely to be poor for individual genetic variants. This is due to the lack of identification of all susceptibility-associated variants, their modes of interaction with each other and the environment. Clinical utility measures the risks and benefits of the genetic test for the management and control of the concerned disease [13]. Population and clinical-based studies are required to evaluate these criteria for a test in diverse populations. T2 research also comprises the evaluation of benefits and risks in a wide range of ethical, legal and social issues (ELSI). As genetics moves into the direction of genomics, and as a genetic test moves into the direction of genome-based health information, it becomes obvious that the ACCE framework is of limited use for evaluation. Instead, the Health Technology Assessment (HTA) approach is already used and has been established as an evaluation tool within the European Member States in the last ten years [5]. This means that the end result of such research is a systematic review and synthesis of pieces of evidence that will support the development of evidence-based policy and practice guidelines [8,14]. The translation of genomics into guidelines requires a novel type of action. New models of HTA are needed that can account for the unique types of evidence inherent to individualised targeted therapies [15]. CTA (constructive technology assessment), for example, is a means to guide early implementation of new developments in society and has been used to evaluate the introduction of a new diagnostic test in The Netherlands, the 70-gene prognosis indicator (Mamaprint^®^) for node-negative breast cancer patients [16].

Phase 3 research, the translation of evidence-based guidelines to health practice is one of the most challenging problems in healthcare and disease prevention [8]. It comprises issues such as increasing the spread of knowledge about evidence-based interventions (dissemination research), integrating these interventions into existing programs and structures (implementation and health services research), and widespread adoption of these interventions by the whole range of stakeholders (diffusion research) [8]. Additional challenges include workforce training, public health literacy, information systems and public participation [17].

The last phase of the continuum of translation research assesses how the adoption of evidence-based recommendations and guidelines can make an impact on real-world health outcomes [8]. T4 research often focusses on clinical and public health outcomes. Additionally, the population health impact should be monitored constantly. Therefore, in different populations there has to be a systematic evaluation of the added value of using genetic information on health outcomes (in terms of attributable risks); the scope and the purpose of using genome-based services; and the quality of the genetic information and counselling services [4,5,8].

Nowadays, there are more than 350,000 published human genetics and genomics articles, but it is estimated that less than 3% deals with translation research T2 and beyond [8]. This means that evidence-based guidelines are very uncommon, and T4 research is even hardly done. Although moving scientific discoveries into policy and practice and the delivery of population-level health benefit has always been slow and difficult at best, many believe that this is a doable project when all relevant stakeholders on all levels are involved [5,18].

## Role of epidemiology

One of the main contributions of genetic epidemiology is to have a more thorough understanding of the causes of complex diseases by taking notice of the genetic contribution to those disorders [4]. To date, many of the proposed advances resulted from genetic epidemiological research made use of methods like the investigation of familial clustering, twin, adoption and migration studies. The specific genetic determinants have been identified through linkage analysis and, more recently, through genome-wide association studies (GWAS). The national institutes of health defined a GWAS as a study of common genetic variation across the entire human genome designed to identify genetic associations with observable traits [19]. These studies use high-throughput genotyping technologies to assay hundreds of thousands of single-nucleotide polymorphisms (SNPs) and relate these genetic variants to diseases or health-related traits [19]. The molecular and cellular processes, which are encoded by the DNA sequence and thus possibly by the considered SNPs, are susceptible to influences from the external environment. Therefore, besides establishing the gene-disease association, studies on the gene-environment interaction must be addressed. Recently, the Gene Environment Association Studies (GENEVA) consortium was initiated to address these issues [20]. One important disadvantage of GWAS is the occurrence of false-positive and false-negative associations. Therefore, the last part of a GWAS should be the replication of identified associations in an independent population sample [19]. Additionally, knowledge of genetic variants can be used to establish the causal nature of environmentally modifiable risk factors, through the application of "Mendelian randomisation" [18]. The basic principle in such studies is that if genetic variants either alter the level of or mirror the biological effects of a modifiable environmental exposure that itself alters disease risk, then these genetic variants should be related to disease risk to the extent predicted by their effect on exposure to the risk factor [18]. Because observational studies are prone to confounding, this can be a more reliable way to address environmental risk factors of disease. Furthermore, molecular epidemiology and systems epidemiology based on basic genomics research, epigenomic patterns and genome-environmental interactions are promising new developments in the field of epidemiology [21].

Although the GWAS often offer robust associations between certain SNPs and complex diseases, the population implication of findings from many such studies are unclear because basic population-based genetic prevalence data are often unavailable [22]. Population-based research is essential to quantify the population prevalence of genetic variants, the magnitude of disease risk associated with these variants (in relative and absolute terms), the contribution of these variants to the occurrence of disease in different populations (attributable risk), the existence of gene-environment and gene-gene interactions and the validity of genetic tests based on such variants in predicting disease risk [10,23]. One of the aims of the HuGE reviews is to promote the completion of reviews, which are peer-reviewed, systematic synopses of the epidemiological aspects of variation in particular genetic variants and health outcomes [11]. A major future challenge is to rethink these approaches in the light of personalised medicine and healthcare: the more evidence is provided on the individual (personal) level, the less evidence can be expected on the population level due to interindividual genomic variation including individual epigenomic effects.

Genetic epidemiology research relies heavily on routine health records such as electronic patient records or disease registries for the identification of disease cases, controls or risk factor information and risk patterns. Whereas the use of routine datasets for genetic research is well established the opposite has not taken place; that is, the contribution of routinely collected data to translate findings from research into population health activities such as surveillance, needs assessment, health promotion and disease prevention programmes and evaluation [24]. Anyhow, epidemiological approaches are fundamental to the population-based biobanks being proposed for gene discoveries and characterisation, as well as to large case-control studies of common diseases using whole genome association analyses [25,26].

The discussion about the relevance of integrating genome-based information into biobanks and about the role of genome-based biobanking for epidemiological research and public health is still in its early stages [26]. In the meantime, various cancer registries and neonatal screening programmes have expanded their existing databases with genotyping to study disease incidence and prevalence, natural history and risk factors. In addition, huge amounts of biological materials are collected to quantify disease incidences in various populations and subpopulations and to gain an insight into the risk factors, including genome-environment interactions. These biobanks are based on large cohort studies (for example in the UK [27]), or even concerned a whole population (for example in Iceland [28]). Nevertheless, the majority of existing biobanks are still relatively small collections of tissue samples related to specific diseases such as cancer.

The future challenges for biobanks with respect to epidemiological research and public health are numerous. They include the incorporation of genomics into existing public health programmes and the integration of genome-based knowledge into future surveillance systems [26,29]. Thus we can explore the use of existing data sources to enhance the contribution of genomics to population health [24]. Possibilities are abundant. One plain example is disease surveillance. Genomics forms an essential part of public health work to combat infectious diseases. Sequencing of pathogens, for example, can allow rapid diagnosis and control of disease. To understand the host response to pathogens and the association of genetic variants with susceptibility or resistance to disease, human genomics research is of great value. But routine datasets are also central to the surveillance of non-infectious disease and here too, the understanding of genomic health determinants as a factor in disease development will be essential. Screening for genetic disease, although by biochemical methods, was already initiated in the early 1960s with phenylketonuria testing and application of the Guthrie test in newborns [30]. Most genetic screening programmes today are limited to single gene and chromosomal disorders and entail different aims like the identification of affected individuals through neonatal or antenatal screening or pre-conceptual testing of couples. Routine databases could also play a role in personalised disease prevention programmes by assisting the identification and targeting of patients in the various screening groups and the management and evaluation of programmes [24]. In the future it could be interesting to link the individual information during the whole life span, with the aid of the electronic patient records [26].

## Personalised medicine

Concrete applications of genomics in public health, especially based on GWAS, are most pronounced in the field of pharmacogenetics and pharmacogenomics [31]. These themes originated by the notion of the existence of large interindividual differences on drug reaction. A certain drug can have a therapeutic effect on some, but can be ineffective in others and certain people show adverse drug effects on a dose which is subeffective by others. Pharmacogenetics is based on the principle that it is time to use the right drug in the right dose in the right patient at the right time using integrated clinical and genomics parameters [32]. One well-known therapeutic example is the selected use of Trastuzumab, only in patients whose breast tumour expresses the her2/neu gene [33]. The recommendation of the FDA to select the starting dose of Warfarin to reduce the risk of thromboembolic events based on CYP2C9 and VKORC1 polymorphisms is another example of a well-introduced application of genomics into clinical practice [34]. Apart from these, the number of examples based on GWAS in pharmacogenomics are increasing (see the recent review of Wang et al [31]). However, although pharmacogenomics acknowledges the central role of the genetic constitution of a patient to drug metabolism, numerous other factors alter drug response including other drugs, environmental and physiological factors such as nutrition, ageing, liver and kidney function and patient compliance, which in the end results in the understanding of individual pathways in systems biomedicine.

Personalised medicine is the translation of the science of pharmacogenomics and other individualised interventions into clinical practice [35]. Personalised medicine involves preventive, diagnostic, and therapeutic interventions, with risk defined through genetics as well as clinical and family histories. An example is genetic testing to determine the risk of breast cancer. Women carrying a certain BRCA1 mutation have a cumulative risk of 65% by the age of 70 years to develop breast cancer [36]. However, currently there are no standard criteria for recommending or referring someone for BRCA mutation testing. Candidates for BRCA genetic testing are identified through careful analysis of the family history, the patient's ethnic group and the pathologic features of the breast cancer [37,38]. Further studies on the genetic hallmarks of breast tumours indicated that early and inherent genetic properties of the tumour entail the tumours' ability to metastasise [39]. A Dutch research group identified a gene-expression profile, based on 70 genes, that turns out to be a powerful predictor of disease outcome in young patients [40]. This will allow clinicians to select patients who would benefit from adjuvant systemic treatment, reducing the rate of both overtreatment and undertreatment [40].

Expectations about the future impact of the discoveries through the GWAS on preventive and clinical health care practice are high. Despite the current euphoria, the predictive value of genetic profiling is still limited for most complex disorders [41]. This is because most individual gene variants associated with common diseases will have low positive predictive value and associated attributable risk [7]. Also, for some known markers much remains to be learned about their contribution to the incidence of disease [42]. For example, homozygotes for the APOE ε4 gene variant suffer from an increased risk of Alzheimer's disease, but many people who test positive for this allele will not develop Alzheimer's disease [7]. In fact, fewer than 30% of people with the APOE ε4 polymorphism develop Alzheimer's disease [43]. Thus, notwithstanding the growing availability of commercial, direct-to-consumer genetic testing via the internet, evidence-based applications of genetic profiling in clinical and public health care practice are still a far future prospect. Nevertheless, current dynamic developments such as in systems biology, epigenomics and the Personal Genome Project (PGP) ask for different approaches having the potential for personalised medicine and healthcare, since they provide insights and evidence on the individual level [2]. Most of the scientific attention should, therefore, be focussed on basic genomic research, unravelling the understanding of common diseases in all its complexity and all its interactions [41].

## Public Health

About the role of genomics in improving public health some scepticism has been raised. Some authors have stated that genomics undercuts efforts to address social and environmental causes of ill health [25]. Some reject genomics research as an unwarranted extension of the individual risk paradigm [44]. The challenge in Public Health Genomics is to reconcile traditional public health concerns, such as equity and access to health for all population groups, with safe, effective health interventions and diagnostics tailored at the level of each individual. To address the role of human genomics in improving population health Khoury et al. defined three priorities [45]: 1) serving as the honest broker for emerging genomic applications in practice; 2) implementing current evidence-based genomic applications to improve health and prevent disease, while discouraging premature use, misuse, and overuse of genomic applications and 3) using genomics tools to evaluate the health impact of public health interventions regardless of whether they currently use genomics.

Population-based data on genome-disease associations and genome-environment interactions form the basis for studying the added value of genome-based health information to existing recommendations for disease prevention. They will help to gain more insight in the contribution of genomic variants to common diseases. Public health agencies must, in the meantime, work out the appropriate methods to collect and monitor the results of genome-based research and technologies to identify information gaps on the population as well as on the individual level and to formulate the policy development of evidence-based strategies in this domain [26].

Systematic evaluation is critical for improving, accounting for and prioritising public health actions involving genomics. Public health must therefore evaluate the impact of using genetic information on morbidity, disability and mortality associated with disease in different populations and individuals [2,4]. Genetic work is mostly undertaken in outpatient departments, either within clinical genetics, or by the specialty concerned with the ongoing clinical care of the patient (for example paediatrics, neurology, cardiology, oncology etc). The need to quantify such activity, including the magnitude and determinants of the utilisation of genetic tests and services, and the quality of genetic tests and clinical and preventive services relative to established standards and guidelines, is vital for needs assessment, review and evaluation of services, and for the quantification of unmet needs and assessment of service inequalities [4,14,24]. Based on these needs, the future challenge for epidemiological research and public health include the incorporation of genomics into existing public health programmes [26]. Furthermore, whereas the concept and use of "genetic tests" may not reflect the complexity of most diseases, much more plausible and practicable seems to be the concept and use of "genome-based information", since it is based on the understanding of genome-environmental interactions changing over time and space within individuals. In the landscape of Public Health Genomics these two different approaches and ways of thinking can already be observed: whereas institutions such as the Public Health Genomics Foundation (PHGF) in the UK or the Institute of Public Health Genetics in Seattle, USA, focus on the role of genetic tests for public health, other institutions such as the Institute for Public Health Genomics in Maastricht, The Netherlands, the European Centre for Public Health Genomics (ECPHG) or the Public Health Genomics European Network (PHGEN) focus on the role of genome-based health information including information from systems biology, epigenomics and the Personal Genome Project for public health.

The potential for genomics to promote public health in case of smoking is a helpful example to illustrate the current usefulness, threats and limitations of these applications. Smoking-induced lung cancer continues to be a major public health concern. However, it is widely known that smoking cessation will lead to a decreased risk of lung cancer and other smoking-related diseases such as chronic obstructive lung disease, coronary artery disease, asthma and other smoking-related conditions [46]. For years, cessation programmes targeted the individual smoker with a limited but tangible success. From a public health perspective, societal-level approaches such as bans, taxes and antismoking campaigns showed a more substantial utility. These methods have a dual benefit: they decrease the smoking prevalence, and in the case of smoking bans, they prevent the exposure of vulnerable populations to secondary smoke. The emerging question today is how genomics could augment those public health effects [47]. First of all, genome research can identify genotypes that modulate smoking status, initiation, cessation, quantity and treatment response. In addition, the discovery of polymorphisms linked to an altered risk of lung cancer would be valuable. Secondly, genetic testing could lead to an increased rate of smoking cessation. The first mechanism Carlsten et al proposed for this is that if a smoker is aware of a higher risk of lung cancer, he or she will have a much greater motivation to stop [47]. Furthermore, genetic testing could identify individuals who should be subjected to more intensive cessation programs. Determining whether these two approaches provide a public health benefit requires attention to two questions. First, how does genomic knowledge improve existing individual or societal interventions? And second, will the augmentation in cessation rates due to genetic testing justify the risks? Some drawbacks and risks associated with genetic testing could be hypothesised too. For example, the knowledge of having an adverse polymorphism could lead to substantial anxiety. The lack of cessation success could be blamed on genetic factors and someone could therefore lose the remaining motivation to quit. The opposite could be true too. If a genetic test shows no increased risk of smoking related diseases, it could be seen as a justification to start or to keep on smoking. And it could serve the tobacco industry as a defence against liability. One could imagine misleading marketing campaigns such as "Is cigarette smoking safe for you? This test can help you decide." This shows that the potential harms are significant. However, there is a motivation to study the genetic components of all smoking aspects. It will lead to a better understanding of the underlying mechanisms of substance abuse and could lead to innovative therapeutics with applicability beyond smoking. Furthermore, it could reveal novel signalling pathways that contribute to the onset of lung cancer and other smoking associated illnesses. Although genomics studies could be acknowledged as currently unlikely to lead to a reduction of lung cancer compared with the proven, non-genetic-based strategies for smoking cessation, our smoking behaviour is not only influenced by social factors, but also by our genomic make-up with its multiple variants. If for example, we try to empower people to stop smoking, we see that there are people for whom it is almost impossible to quit, and today we know that to a large extent this may be due to genomic variants that specifically predispose to nicotine addiction [47,48].

### Population-based versus high-risk-group-targeted strategies

Public health aims at improving the health of the entire population by implementing preventive strategies. Of course, in the end, behind the entire population is the individual. The progress made in genomic research of common diseases increases the interaction and the interdependence between the traditional healthcare system and the public health system. In other words, the interaction between focussing on treatment of individuals and on prevention and control in populations is growing. One of the main issues of PHG is how to reconcile the notion of increased personalised medicine and healthcare with public health interventions at population level. This distinction was first set out by Geoffrey Rose who, however, was careful to present these approaches as complementary rather than mutually exclusive [49].

One of the promises of individualised medicine is the possibility of engaging in a level of preventive care that far exceeds current abilities. Screening programmes are, by their very nature, inefficient because an entire population is subjected to screening while relatively few individuals benefit and some are actually harmed. This inherent inefficiency is expensive for both the individual (in terms of morbidity) and for society (in terms of costs) [50]. Thus, there is the potential for more target-oriented and stratified prevention strategies to finally replace the 'one size fits all' approach [51]. This is also reflected in one of the general objectives of the first phase of the Public Health Genomics European Network: the sharing of experience in health risk reduction policies aiming at applying stratified strategies instead of 'one strategy for all' [52]. Thus, there is the possibility to avoid ineffective preventive strategies. There is already the challenge to differentiate between persons, who will respond to certain vaccinations and those who will not. The recommended BCG vaccination is a remarkable example. In children with an interferon-gamma receptor deficiency, this vaccination can cause a disseminated BCG infection which may be lethal [53].

The ultimate goal of public health genomics is, once we understand both the genetic and environmental factors involved in the causation of disease, and how they interact, to devise effective preventive interventions targeted at individuals with specific genotypes [54]. The field of epigenomics is expected to contribute a lot to this goal. Its main attempt would thus not concern the new technological advances such as gene therapy but subcategorising populations compatible to the effectiveness of preventive environmental interventions stratified according to their genetic risk. The rationale behind this strategy is that the molecular and cellular processes, which are encoded in the gene sequence, are susceptible to influences from the external environment [54]. Anyhow, the genetic association studies will likely lead to population-wide health protection and disease prevention efforts, and not only to targeted interventions based on genetic susceptibility [55].

Taking these new developments into account, the assurance of the complementary nature of individualised and population-oriented interventions within the healthcare systems, which has already been stressed by Geoffrey Rose decades ago, appears to be even more valid than ever before. Nevertheless, there is a huge paradigm shift for public health. Whereas prevention strategies will turn more and more towards secondary prevention and primary prevention might become a questionable concept, health protection and health promotion messages will be even more general. In the end, the task of public health is to assure both strategies on the population level. The major shift is that both strategies can only be effective if they start with looking at the individual first, behind whom might be a subpopulation and even the whole population. That means that our current public health approach looking at the population first and then dividing it into subpopulations might be obsolete very soon.

## Multidisciplinary character of PHG

Public health genomics is an interdisciplinary research field which tries to bring together different disciplines like the medical sciences, statistics, biotechnology and engineering, pharmaceutical research and industry, policy writing departments, ethics, law, sociology, public health practitioners, genetic centres, governments, representatives of patient groups etc. One of the challenges in the evaluation of genomic applications to healthcare is the integration of studies of the normative, legal economic and governance implications (ELSI). These ethical, legal and social issues are somewhat arbitrary categories, because these issues are almost always intertwined. As cornerstones of ethical medical research practice the fundamental rights of the individual, such as privacy issues and the person's autonomy, deserve unbiased attention in PHG too. However, the public health perspective asks for principles going beyond the development of responsible health policies and frameworks (PHELSI). It must also be considered that information of the individual genome will harbour information of the genetic constitution of the parents and indirectly from other family members too. This raises again issues of confidentiality, privacy and the 'right (not) to know' [5,56]. Indeed, feedback of findings is an important part of building and maintaining public trust in research [57]. Feeding back research results from genomics studies is complicated by a number of factors. The most significant one is that a GWAS is a research tool and is not designed for clinical diagnosis [57]. Although the clinical value of the genome in general will expand, many of the discoveries of whole genome methods currently identify genetic variants that are responsible for very small increases in disease risk. In such situations, there is a need for management pathways to determine whether or what information is fed back, who is responsible for feeding back results, and whether this also extends to other family members [57,58]. In general, the results offered should be scientifically valid, confirmed, and should have significant implications for the subject's health and well-being [58].

Another privacy issue is related to biobanking [26]. To date, biobanking and public health surveillance have been looked at independently from each other [59], resulting in the fact that existing databases, created for other purposes, are not that easy to extend with genomic information. In the case of genomic biobanking, it will, for example, not be possible to render data completely anonymous because it requires an ongoing contribution of the research subjects and the linkage of old and new information. This brings along the problem of consent. Concerning existing databases, the involved individuals, families and communities or populations may not have explicitly consented to (future) studies on the relationship between the human genome and susceptibility to the disorders being studied in that particular database [59].

In this light, (digital) data sharing becomes a potential problem, too. Scientific policies tend more and more to incorporate open access guidelines. This raises a number of ethical issues as many of the principles and procedures in medical research are not designed for wide-scale data sharing or public health purposes [57]. Therefore, the form of informed consent should be updated [58]. The enrolment of study participants should start with a proper counselling session, which requires a thorough training for physicians and other health professionals. The consent form should, for example, contain information about the return of data and the possible implications for family members [58]. Also the ability to withdraw from subsequent studies must be well considered, especially when the data are released into a publicly accessible database [58]. This all means that policy makers must be aware of the current challenge to improve consumer protection and to monitor the implications of genome-based knowledge and technologies for health, social and environmental policy goals [4]. Furthermore, it might be necessary to rethink our current governance frameworks in the light of the new developments in genomics [60,61].

There still is a discussion about stigmatisation and discrimination due to the predictive power of genetic information [56]. Although the idea of 'genetic determinism' and the 'geneticalisation of society' are obsolete [62], the most commonly expressed fear is that genetic information will be used in ways that could harm people [63]. It also has to be stressed that public health genomics has nothing to do with modifying genes. A genetic marker is 'just' another modifier to explain the likelihood of developing a disease. It deserves no 'status apart' just because it was gained with the aid of molecular diagnostic techniques [56,62]. However, it should be emphasised that genetic information should be considered as a highly sensitive group of medical information [5]. In the end, with the use of predictive genetic tests, it will turn out that 'we are all at risk for something'. This involves the first potential for social inequalities in health, namely the accessibility of the tests, if the genetic tests are not reimbursed by the health insurances, which will lead to a two-tire system [62]. Another source of social inequality or discrimination will depend on the degree of understanding and genomic literacy, that is being able to make sound decisions about the risk calculation by the common population [62]. To return to the example of smoking, it might be possible that healthcare systems focus efforts only on those at highest risk of developing smoking-related disorders and neglecting others. Physicians might be less motivated to encourage cessation in those smokers who have a genetic profile that predicts poor response to multiple treatment modes [47]. This is what has been called the "double-edged sword" of genetics, because it can be considered that implementing genomics in public health can result in either the widening or the narrowing of health disparities among the population.

The major factor that determines the rate of evolution is the social context and the degree to which society and individual citizens concur with the research agenda [64]. A systematic review concerning the current information of genetic health services for common diseases identified that consumers have vague and divergent notions about the value of genetic testing for common disorders [65]. The same review indicated that the primary care workforce, that will be required to be on the front lines of the integration of genomics into practice, feels underprepared to do so. Their inability to provide appropriate counselling for their patients is due to this lack of basic knowledge about genetics and their lack of confidence in interpreting familial patterns of disease [65]. A 2003 report by the Institute of Medicine identified genomics as a priority for the training of all public health professionals in the 21^st ^century [66]. Therefore, the public health community has a major role to play in raising the level of general genomic literacy, developing targeted messages about the uses of genetic information in disease prevention and coordinating communication strategies with stakeholder groups [4]. Craig Venter's research group already called for the scientific communities to be in continual conversation with the entire society [67]. And again, new developments in genomics such as derived from systems biology, epigenomics and the Personal Genome need to be taken into account in order to be able to truly benefit from genomics information as part of health information. Furthermore, individuals now have access to their own genomes through direct-to-consumer genetic testing companies. These events outside medical research cannot be ignored, as there is the potential for them to have an effect on the medical research context, which in the case of an unfavourable event could undermine the public trust and support that is necessary for medical research to continue and to thrive. Thus, the challenges we are facing should be considered in the light of events occurring in the broader social context [57].

An underdeveloped topic in public health ELSI (PHELSI) discussions is how medicine and public health might approach ethical questions differently. Therefore, Thomas et al. [68] applied the 12 principles of the public health code of ethics to genomics. Ethical questions in public health often address the tension between the right of individuals and the good of the overall community, democratic processes in determining health policies, the prevention of conditions that lead to poor health, and the protection of disenfranchised or marginalised populations. Bioethics appears to have a sound and systematic foundation: it rests on the four pillars of principlism (beneficence, non-maleficence, autonomy and justice) [67]. Placing principlism's thinking in a larger perspective, the broadest and most important question is whether the public's health is actually improved by the knowledge derived. This brings us back to the phase 2 translational research as pointed out above, again indicating that all issues involving the introduction of genomic knowledge into public health practice are intertwined.

To conclude, in order to succeed, the competing interests of different stakeholders have to be managed and reconciled in a collaborative framework that rewards both scientific innovations as well as appropriate clinical and public health applications. In the short run, we need to develop a policy framework that imposes accurate laboratory testing, and truth in advertising about potential health benefits, protection from untoward psychological or social effects, as well as reimbursements of genome-based services that can benefit individuals, families and populations [55].

## Conclusion

Today, the mounting accomplishments of the Human Genome Project and further developments in genomics demand that we rethink the role of genomics in every condition of public health interest. This requires a conceptual shift in both public health and medicine [5]. As a consequence, Brand et al. described a dichotomy: genomics needs to understand how it can include public health aspects in its work programme while public health needs to analyse how genomics changes the concepts of public health. As the name implies, the second approach is seen as the core task of PHG [5].

Many papers spelled out prospects and major benefits of genomic medicine even in the near future [42]. However the extent to which this increased understanding will lead to measurable improvements in human health is as yet unclear but that it is likely to do so sounds very plausible. The unknown factor is the time scale of such advances [64]. An important and early role for PHG will be to counter the hype that accompanies much of the genome-based research. In addition, it has to stress the importance of ensuring that any new test or intervention arising from genomics research is not introduced too soon but is comprehensively evaluated and supported by evidence [54]. However, Khoury et al. estimated that nowadays less than 3% of the research deals with translation phases T2 and beyond [8].

The greatest potential public health benefit from genomic profiling lies in improving common disease prevention [18]. However prospects for this type of prevention are few. The main problem is that very few common genetic variants are known to increase risk of common diseases substantially [18]. Even when a genetic risk is substantial, knowledge of the risk contributes to improved health outcomes only if effective measures are available for preventive or early treatment [7]. Similarly, genetic testing is of uncertain value when the available interventions are not genotype-specific. Furthermore, for a genetic test to be useful in the management of a common disease, it must have predictive powers over and above accepted risk factors which can easily be measured [18]. Genetic testing will have its greatest public health value when it identifies individuals who would benefit from specific interventions based on their risk [7,54]; and especially when this risk is understood as a risk pattern based on individual genome-based health information instead of the narrowed idea of a genetic test.

Ultimately, the translation of advances in genome research to routine patient care will require an educated population and educated healthcare professionals. This barrier should not be underestimated [42]. Therefore, the impact of genomics in the future calls for a strategy to develop a public health workforce that is adequately prepared. Thus, a major goal for the public health institutions should be to create an infrastructure that supports implementation and evaluation of genome-based activities, dissemination of genome-based health information and education [23]. Cho already pointed out in 1999 that exactly because it was only the development stage of genomic research, there is a valuable opportunity to lay out and refine relevant ethical issues [69]. Although we are twelve years later there is still a need for more empirical methodologies so that the available bioethics and policy frameworks recognise that one size will not fit all, and that the context in which a candidate genomics application is used also matters. Policy or other interventions should clearly define the issues that are raised and ought to be targeted when addressing the bioethics impact.

## Conflict of interest statement

The authors declare that they have no competing interests.

## Authors' contributions

EC reviewed the literature, participated in the conception of the study and drafted the manuscript. JV participated in the conception of the study, critically revised the manuscript and coordinated the final version. AB and HV critically revised the manuscript and provided additional information. All authors have read and approved the final manuscript.
